# The Color Puce (*Pyüs*)

**DOI:** 10.3201/eid2802.212274

**Published:** 2022-02

**Authors:** Clyde Partin

**Affiliations:** Emory University, Atlanta, Georgia, USA

**Keywords:** puce, the color of puce (*pyüs*), fleas, vector-borne infections, plague, bubonic plague, color of fleas, synesthesia, Marie Antoinette

## Puce [pyoos]

For those with synesthesia, in whom stimulating 1 sensory pathway gives rise to a subjective sensation of a different character, the word plague may chromatically resonate with puce (Figure). In pre-revolutionary France, an era of “evocative color nomenclature,” Marie Antoinette’s reign was precipitating intense criticism. Her countrymen were experiencing severe socioeconomic stress, thus her sartorial self-indulgence was much resented.

**Figure:**
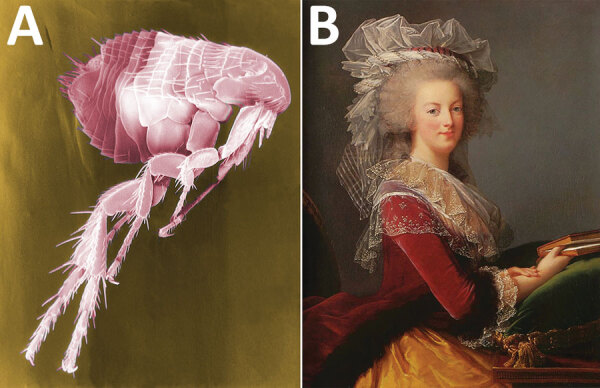
A) Digitally colored scanning electron microscopic image of a flea. Puce is a particularly difficult color to describe. Numerous shades of puce exist, some of which are associated with different anatomic areas of the flea. Image no. 11436: Janice Haney Carr/CDC. B) Portrait of Marie Antoinette painted in 1785 for the Ministry of Foreign Affairs, by Louise Élisabeth Vigée Le Brun. Private collection, Public domain. She seemed to have “preferred a shade leaning more toward ash-gray,” but is seen here modeling a more standard hue of puce.

After discovering the Queen wearing a new gown, her husband, Louis XVI, the King of France, chided her, describing the dress’s unflattering purple‒brown hue as “*couleur de puce*” (color of fleas). This admonishment had the unintended consequence of promoting puce as the exclusive color worn by the French court. Puce, the French word for flea, descends from *pulex* (Latin). Flea droppings leave puce colored “bloodstains” on bedsheets. The role of fleas, however, as a vector for bubonic plague was not proven until about 1895. 
